# Evaluating surgical and non-surgical wound approaches in diabetic foot ulcer treatments: A comparative study

**DOI:** 10.6026/973206300223300

**Published:** 2026-06-30

**Authors:** Ashis Tiwari, Pradeep Rathore, Ashish Kumar Shukla

**Affiliations:** 1 Department of General Medicine, Government Medical College, Datia, Madhya Pradesh, India; 2 Department of General Surgery, Government Medical College, Datia, Madhya Pradesh, India

**Keywords:** Surgical and non-surgical wound, diabetic foot ulcer (DFU), infection

## Abstract

Diabetic foot ulcers (DFUs) remain a serious complication of diabetes mellitus, often leading to infection, prolonged hospitalization and potential limb amputation. Therefore, it is of interest to evaluate the outcomes of surgical versus non-surgical management of DFUs in 100 patients, divided equally into two groups of fifty each. Group A received surgical interventions such as debridement and minor procedures, while Group B underwent non-surgical care including dressings, antibiotics, glycemic control and off-loading. Healing progress, infection control, duration of recovery and complication rates was closely monitored. Thus, we show that surgical management yields faster healing and superior infection control in moderate to severe cases, while non-surgical care is suitable for mild ulcers, advancing current understanding of tailored treatment strategies to optimize DFU outcomes.

## Background:

Numerous chronic consequences impacting several organ systems are linked to diabetes mellitus, making it a significant worldwide health issue. One of the most severe and incapacitating problems among these consequences is diabetic foot ulcer (DFU). A foot ulcer is a major cause of hospitalization and lower limb amputation among diabetes adults, affecting an estimated 15-25% of patients during the lifetime [1, 2]. Damage to tissues and a slowed healing process are the main causes of diabetic foot ulcers, which may be attributed to peripheral neuropathy, peripheral vascular disease and an altered immune response [3]. Multiple factors contribute to the complicated pathophysiology of diabetic foot ulcers. The danger of unrecognized trauma and repeated stress injuries increases when peripheral neuropathy causes a decrease in protective feeling in the foot [4]. Furthermore, peripheral artery dysfunction leads to vascular insufficiency, which in turn reduces tissue perfusion and hinders the supply of oxygen and nutrients needed for wound healing [5]. Hyperglycemia further contributes to impaired leukocyte function, increased susceptibility to infection and delayed wound healing processes [6]. Consequently, diabetic foot ulcers frequently become chronic wounds that are difficult to manage and often require multidisciplinary care. Nearly 20% of hospital admissions due to diabetes are diabetic foot problems, which put a heavy strain on healthcare systems worldwide [7]. Lower limb amputation is two to three times as common in persons with diabetic foot ulcers as in non-diabetic people [8]. Early diagnosis and effective management are therefore critical to prevent infection, limb loss and mortality. Management strategies for diabetic foot ulcers broadly include non-surgical (conservative) and surgical approaches. Non-surgical management typically involves wound care with appropriate dressings, strict glycemic control, antibiotic therapy for infection, pressure off-loading and patient education [9]. These measures aim to promote wound healing by controlling infection, improving circulation and minimizing mechanical stress on the affected area. Conservative wound management remains the first-line treatment for mild to moderate ulcers and is often effective when implemented early [10]. However, in cases where ulcers become infected, necrotic, or fail to respond to conservative therapy, surgical interventions may become necessary. Surgical approaches include procedures such as debridement, drainage of abscesses, minor amputations and reconstructive procedures aimed at removing necrotic tissue and promoting a healthy wound environment [11]. Surgical debridement, in particular, is widely regarded as an essential component of DFU management as it helps eliminate devitalized tissue and reduce bacterial load [12]. Several studies have reported improved healing outcomes and reduced risk of complications when surgical interventions are appropriately integrated into the treatment plan [13]. Despite advances in wound care and surgical techniques, the optimal management strategy for diabetic foot ulcers remains a topic of ongoing research. The choice between surgical and non-surgical treatment largely depends on the severity of the ulcer, presence of infection, vascular status and patient comorbidities [14]. Therefore, it is of interest to examine diabetic foot ulcers and determine if surgical or non-surgical wound treatment methods were more beneficial, with the aim of identifying treatment strategies that improve healing outcomes and reduce complications.

## Materials and Methodology:

## Study design:

The present study was conducted as a prospective comparative study to evaluate the effectiveness of surgical and non-surgical wound management approaches in patients with diabetic foot ulcers.

## Study setting:

The study was carried out in the Department of General Surgery at a tertiary care hospital over a period of 12 months.

## Sample size:

A total of 100 patients diagnosed with diabetic foot ulcers were included in the study.

## Study groups:

The participants were divided into two equal groups:

[1] Group A (Surgical Management Group): 50 patients managed with surgical interventions such as wound debridement, drainage of abscess, or minor surgical procedures when required.

[2] Group B (Non-Surgical Management Group): 50 patients treated with conservative methods including wound dressing, antibiotics, glycemic control and off-loading techniques.

## Inclusion criteria:

[1] Patients aged 18 years and above with diagnosed diabetes mellitus.

[2] Patients presenting with diabetic foot ulcers of varying severity.

[3] Patients who provided informed consent to participate in the study.

## Exclusion criteria:

[1] Patients with non-diabetic foot ulcers.

[2] Patients with critical limb ischemia requiring major amputation.

[3] Patients with severe systemic illness or malignancy.

[4] Patients unwilling to participate in the study.

## Data collection:

Detailed patient information including age, gender and duration of diabetes, ulcer size, location, infection status and associated comorbidities was recorded. Clinical examination and relevant laboratory investigations were performed to assess the severity of the ulcer and overall health status of the patient.

## Treatment protocol:

Patients in the surgical group underwent appropriate surgical procedures such as debridement or drainage, followed by regular wound care and antibiotic therapy. Patients in the non-surgical group received standard wound care management, including antiseptic dressings, systemic antibiotics when required, glycemic control and pressure off-loading techniques.

## Outcome measures:

The primary outcomes assessed during follow-up included:

[1] Rate of wound healing

[2] Time required for ulcer healing

[3] Control of infection

[4] Occurrence of complications

[5] Need for further surgical intervention

## Follow-up:

Patients were followed up at regular intervals until wound healing or significant clinical improvement was observed.

## Statistical analysis:

Microsoft Excel was used for data entry and SPSS (version 26.0; IBM Corp., Armonk, NY, USA) was used for statistical analysis. We used descriptive statistics like percentages, standard deviation and mean to summarize the data. A p-value less than 0.05 were deemed statistically significant when doing comparative analysis between groups using the Chi-square test and Student's t-test.

## Results:

This comparative research comprised one hundred participants who had diabetic foot ulcers. Two groups were formed from the participants: Group A, which dealt with surgical wound care (n=50) and Group B, which dealt with non-surgical wound treatment (n=50). Various clinical and outcome parameters were analyzed to assess the effectiveness of the two treatment approaches. Patients between the ages of 51 and 60 made up the bulk of both groups; this was true in the surgical group (36.0%) and the non-surgical group (30.0%). The surgery group included 28.0% patients aged 41-50, whereas the non-surgical group had 32.0% patients of the same age. People in the surgery group were 20.0% older than the average, while those in the non-surgical group were 18.0% older; individuals in the 30-40 age bracket made up 16.0% and 20.0% of the total, respectively. There was no statistically significant variation in the age distribution between the two groups (p = 0.82), suggesting that both groups were similar in terms of demographic features (Table 1). The current research found that whilst 54.0% of patients in the non-surgical group did not have full wound healing, 72.0% of patients in the surgical care group did. Twenty percent of surgery patients and thirty percent of non-surgical patients showed signs of partial healing; eight percent of surgical patients and sixteen percent of non-surgical patients showed no improvement. Compared to conservative therapy, surgical wound care showed a greater percentage of full healing, according to the data. Surgical surgery improved healing results in individuals with diabetic foot ulcers, as shown by the statistically significant difference between the two groups (p = 0.04) (Table 2). The results showed that infection control was achieved in 76.0% of patients treated with surgical wound management, whereas 60.0% of patients in the non-surgical group achieved infection control. Conversely, persistent infection was observed in 24.0% of surgical patients and 40.0% of those receiving non-surgical treatment. Surgical intervention, namely debridement and removal of necrotic tissue, seems to be more efficient in limiting infection in diabetic foot ulcers, according to the statistically significant difference between the two groups (p = 0.03) (Table 3).

## Discussion:

An important source of lower-limb amputation, disability and hospitalization on a global scale, diabetic foot ulcers (DFUs) are a consequence of diabetes mellitus. Wound healing, infection prevention and complication reduction may all be achieved with the use of effective treatment measures. In this randomized controlled trial, researchers looked at 100 diabetic foot ulcer patients and compared the results of surgical and non-surgical wound treatments. The results demonstrated that surgical intervention showed better healing outcomes and infection control compared to non-surgical treatment, although conservative therapy remains beneficial in selected cases. In the present study, complete wound healing was observed in 72% of patients in the surgical group compared to 54% in the non-surgical group. These findings are consistent with previous studies that reported higher healing rates following surgical management such as debridement and revascularization. A systematic review reported that surgical treatment achieved wound healing rates of approximately 84%, whereas non-surgical approaches achieved around 60% healing in patients with diabetic foot ulcers [2]. Surgical removal of necrotic tissue promotes the formation of healthy granulation tissue and reduces bacterial load, thereby accelerating wound healing. Another important finding in the present study was the better infection control in surgically treated patients (76%) compared to those receiving non-surgical treatment (60%). Infection is a common complication in diabetic foot ulcers due to impaired immunity and poor vascular supply. Early surgical debridement helps remove infected and necrotic tissues, preventing the spread of infection. Studies have shown that osteomyelitis and severe infections occur in nearly 20-70% of diabetic foot ulcer cases, making timely surgical intervention an essential component of treatment [1]. Similarly, a comparative analysis by Piaggesi *et al.* reported healing rates of 86.3% in surgical management compared to 75% in non-surgical therapy, highlighting the effectiveness of surgical approaches in promoting wound recovery [3]. In addition, Aslam *et al.* demonstrated that surgical treatment resulted in a healing rate of 95.5%, whereas conservative management achieved 70.2% healing, further supporting the benefits of surgical interventions in complex ulcers [4]. Nevertheless, when it comes to managing diabetic foot ulcers, conservative therapy is still crucial, especially in the early stages or when the ulcers are not too serious. Optimal conditions for wound healing may be achieved by non-surgical care strategies such as off-loading, antimicrobial therapy, proper wound dressing and tight glycemic control. Studies have shown that standard conservative therapy leads to ulcer closure in approximately 30-40% of patients within 12 weeks [6]. Uppin *et al.* also reported that surgical intervention provided faster source control for advanced or infected diabetic foot ulcers, while non surgical care achieved comparable healing for less severe wounds; thus treatment should be individualized pending prospective confirmation [15]. Although the healing rate may be lower than surgical treatment, conservative therapy carries fewer procedural risks and remains the first-line management for mild ulcers. The findings of the present study also highlight the importance of a multidisciplinary approach in diabetic foot care. Effective management requires collaboration between surgeons, endocrinologists, wound care specialists and infectious disease experts. Multidisciplinary care has been associated with reduced amputation rates and improved clinical outcomes in patients with diabetic foot ulcers [5]. Early identification of risk factors such as peripheral neuropathy, vascular insufficiency and infection can significantly improve treatment outcomes. Advanced wound treatments, such as negative-pressure wound therapy and advanced dressings, are an additional component of diabetic foot ulcer care. Wound healing results may be greatly improved by negative-pressure wound treatment, according to recent clinical investigations. Complete ulcer closure was seen in 87% of patients treated with negative-pressure treatment, compared to 29% in traditional wound management, according to one randomized research [7]. Such advanced therapies may be used in conjunction with surgical or conservative management to improve healing outcomes. Despite the advantages of surgical treatment, it is important to recognize potential complications. Surgical interventions may be associated with higher risks of amputation and postoperative complications in certain cases. For example, some studies have reported amputation rates of approximately 19% following surgical interventions in severe diabetic foot ulcers [1]. Patient education regarding proper footwear, foot hygiene and glycemic control plays a crucial role in preventing ulcer recurrence. Overall, the findings of the present study align with previous research indicating that surgical management provides faster healing and better infection control in moderate to severe diabetic foot ulcers, while conservative treatment remains effective in early-stage ulcers. A combination of both approaches, depending on ulcer severity and patient condition, is often necessary to achieve optimal outcomes.

## Conclusion:

We show that surgical wound management was more effective than non-surgical treatment in achieving faster wound healing and better infection control in patients with diabetic foot ulcers. However, conservative management remains beneficial for mild ulcers and in patients unsuitable for surgery. Early diagnosis is needed to enhance healing results and decrease the likelihood of complications and amputations in patients with diabetic foot ulcers. Thus, it is crucial to use an effective therapy selection strategy that involves a multidisciplinary approach.

## Figures and Tables

**Table 1:** Demographic characteristics of study participants

**Age Group (years)**	**Surgical Group (n=50)**	**Non-Surgical Group (n=50)**	**p-value**
30-40	8 (16.0%)	10 (20.0%)	
41-50	14 (28.0%)	16 (32.0%)	
51-60	18 (36.0%)	15 (30.0%)	
>60	10 (20.0%)	9 (18.0%)	0.82

**Table 2:** Wound healing outcome in surgical and Non-Surgical groups

**Wound Healing Outcome**	**Surgical Group (n=50)**	**Non-Surgical Group (n=50)**	**p-value**
Complete healing	36 (72.0%)	27 (54.0%)	
Partial healing	10 (20.0%)	15 (30.0%)	
No improvement	4 (8.0%)	8 (16.0%)	0.04

**Table 3:** Infection control and complication rates

**Outcome**	**Surgical Group (n=50)**	**Non-Surgical Group (n=50)**	**p-value**
Infection controlled	38 (76.0%)	30 (60.0%)	
Persistent infection	12 (24.0%)	20 (40.0%)	0.03
